# 3D Perception-Based Adaptive Point Cloud Simplification and Slicing for Soil Compaction Pit Volume Calculation

**DOI:** 10.3390/s26103150

**Published:** 2026-05-15

**Authors:** Chuang Han, Jiayu Wei, Tao Shen, Chengli Guo

**Affiliations:** 1 The School of Measurement, Control Technology and Communication Engineering, Harbin University of Science and Technology, Harbin 150080, China; hanchuang@hrbust.edu.cn (C.H.); 2320600039@stu.hrbust.edu.cn (J.W.); 2 Sunny Group Co., Ltd., Yuyao 315400, China

**Keywords:** 3D point cloud, adaptive point cloud thinning, computer vision, error analysis, subgrade engineering, volume measurement

## Abstract

In the field of subgrade compaction quality assessment, accurate volume measurement of excavated pits is hindered by non-uniform point cloud distribution, environmental noise interference, and complex irregular boundary features. To address these challenges, this paper proposes a robust volume detection framework that integrates adaptive point cloud refinement and morphological discrimination. First, a pose normalization method employing RANSAC plane fitting and rigid body transformation corrects the spatial orientation of the raw point clouds. To balance data redundancy removal with feature preservation, a gradient adaptive simplification strategy based on local density feedback and K-nearest neighbor estimation is developed. Subsequently, a cross-sectional area calculation model utilizing piecewise-cubic polynomial fitting is proposed to mitigate boundary noise and accurately reconstruct irregular contours. Furthermore, a dynamic outlier removal mechanism based on the Median Absolute Deviation (MAD) and sliding windows is introduced to eliminate non-physical geometric fluctuations. Finally, the total volume is aggregated using a hybrid strategy of Simpson’s rule and a frustum compensation operator. Experimental results on simulated pits with typical topological defects demonstrate that the proposed algorithm outperforms traditional methods, achieving an average relative volume error of less than 0.8%. This approach significantly improves the robustness and precision of sensor-based automated subgrade compaction quality measurement.

## 1. Introduction

In the fields of 3D spatial perception and digital modeling, high-precision volume quantification of excavated pits in scenarios such as subgrade compaction measurement [1] is a critical component to achieve fill quality assessment and closed-loop control of construction parameters. Traditional volume measurement methods, such as the sand replacement method or water bag method [2], are essentially manual-dependent destructive testing. Such methods not only have extremely low sampling density but are also further limited by random errors introduced during operation, making it difficult to effectively characterize the fine topological features associated with irregular surface fluctuations and local damage on the subgrade pit walls. With the evolution of Light Detection and Ranging (LiDAR) and 3D scanning technology [3], using high-fidelity 3D point cloud data for non-contact spatial information reconstruction has become an important technical approach to improve the digital management of subgrade compaction quality.

Recent studies on road-surface reconstruction and defect quantification have increasingly adopted mobile laser scanning (MLS), drone-based photogrammetry, and image-based 3D reconstruction methods for pavement monitoring. MLS systems provide efficient large-scale acquisition capabilities and are widely used for road condition mapping; however, their spatial resolution is often insufficient for accurately reconstructing localized defects with complex geometries. Drone photogrammetry offers flexible acquisition and large-area coverage but may suffer from illumination sensitivity, incomplete depth reconstruction, and occlusion-related information loss in deep pit structures. These limitations motivate the exploration of close-range high-precision acquisition strategies for localized volume estimation tasks.

However, due to the coarse pit surface texture, shadow occlusion caused by the pit’s depth-to-diameter ratio, and light interference in the scanning environment, the acquired pit point clouds often exhibit non-uniform features: the upper edges contain sparse points mixed with dense noise, whereas the bottom region contains redundant data caused by viewpoint overlap. Existing point cloud simplification algorithms [4] struggle to balance the preservation of fine morphology on subgrade walls with redundant data removal. This leads to geometric distortion in subsequent slice fitting and model reconstruction, directly affecting the robustness of volume estimation required for compaction evaluation.

The proposed framework is primarily intended for localized high-precision defect quantification rather than routine large-scale pavement monitoring. Potential application scenarios include repair-material estimation, airport pavement inspection, racetrack maintenance, and localized engineering quality assessment. In these contexts, accurate volumetric reconstruction and local geometric fidelity are prioritized over rapid large-area acquisition efficiency.

To address the characteristics of non-uniform point cloud distribution and complex boundary morphology in small pits, this paper proposes a volume detection algorithm that integrates adaptive thinning and morphological discrimination. The main contributions of this paper are as follows:

- An adaptive simplification algorithm based on local density feedback: addressing the point cloud density caused by the pit aspect ratio (depth-to-diameter ratio), an adaptive simplification model based on K-neighborhood [5] is constructed. While removing redundant data, the model achieves high-fidelity preservation of key feature areas, such as pit edges and irregular protrusions, through normal vector change rate constraints [6].
- A pit volume reconstruction model based on multi-feature fusion: combining the slicing method [7] with an improved integration strategy, this model effectively resolves volume calculation deviations for asymmetric pits under complex working conditions, significantly improving the precision and real-time performance of subgrade compaction measurement.
- Robust regional segmented area fitting and volume reconstruction model: Considering the asymmetry of the pit wall, a cubic polynomial segmented fitting model based on the least squares criterion [8] is constructed, and the median absolute deviation (MAD) [9] is utilized to dynamically process outlier area values. Combined with Simpson’s rule and a frustum compensation operator, high-precision volume aggregation for finite layered point clouds is achieved. This integrated strategy effectively resolves volume calculation deviations for asymmetric pits, significantly reducing the truncation error and achieving an average relative error below 0.8%.

## 2. Related Work

With the development of optoelectronic detection technology, non-contact 3D measurement technologies based on LiDAR and structured light have gradually replaced physical measurement as research hotspots. Currently, mainstream volume calculation algorithms are divided into two categories: surface reconstruction methods [10] and slicing integration methods. In terms of surface reconstruction, widely adopted technologies include mesh generation algorithms based on Delaunay triangulation [11,12], Convex Hull algorithms [13], and Alpha-Shape algorithms [14]. For example, some studies use Delaunay triangulation networks for topological connectivity of discrete point clouds to close the surfaces of holes. Nevertheless, this method is extremely sensitive to point cloud noise—common noise such as gravel reflections or dust in subgrade pits easily causes erroneous triangular facets. Although the Alpha-Shape algorithm optimizes edge contour extraction to some extent by introducing a rolling ball radius parameter, it faces challenges with non-convex structures like subgrade pits—characterized by a “wide-top and narrow-bottom” shape and severe occlusion in the depth direction. A single parameter cannot balance detail preservation at the bottom and pore filling at the top, which easily leads to the “necking” phenomenon and systematic deviations in volume calculation.

To overcome these limitations, this paper adopts a cross-section integration approach. This type of method reduces the complexity of the 3D volume problem by slicing the point cloud along the depth direction, calculating the cross-sectional area of each layer, and accumulating the results layer by layer. Classical algorithms typically adopt an equidistant sampling strategy combined with the trapezoidal rule or Simpson’s formula for calculation. Although the slicing method is more suitable for the axial characteristics of pits, existing technologies primarily face two major unresolved challenges [15]: first, the fixed-step slicing strategy lacks adaptability, wasting computational resources where pit walls vary smoothly while losing key morphological information at the bottom of the pit or at abrupt feature changes where stones protrude; second, current cross-sectional area calculations are mostly based on simple geometric fitting and lack robust statistical processing for outlier noise, leading to calculation results that often include false volumes caused by multipath reflections [16].

To overcome the limitations of existing technologies in handling non-uniform point clouds and complex topological features, this paper proposes a volume detection algorithm that integrates adaptive thinning and morphological discrimination. Distinct from traditional equidistant slicing or global mesh reconstruction, the algorithm in this paper first utilizes K-nearest neighbor (KNN) local density estimation to construct an adaptive simplification strategy [17], dynamically adjusting the sampling step size based on the spatial distribution density of the point cloud to effectively eliminate redundant data while preserving high-frequency features at pit edges and transitions. On this basis, combined with RANSAC plane fitting for pose correction [18], and introducing morphological denoising based on eigenvalue analysis and a cubic polynomial piecewise fitting model, the improved Simpson’s integration formula is ultimately utilized to achieve high-precision and robust calculation of irregular subgrade pit volumes.

To ensure the precision and consistency of data acquisition, this study adopts a high-precision handheld laser scanning system as the core sensing unit.

Although vehicle MLS performs well in macroscopic road mapping, it is often difficult to capture sub-millimeter level feature details when dealing with local and small geometric deformations such as roadbed compaction pits due to scanning distance and angle limitations. Therefore, this study chose a close range handheld scanning system to provide a high-precision digital alternative to the sand filling method in roadbed quality inspection. The proposed framework is not intended to replace large-scale road monitoring workflows, but to support high-precision local volume estimation in engineering scenarios that require accurate measurement of fill materials, ensuring that the absolute error of pit volume measurement is controlled within the minimum range allowed by engineering specifications.

Compared with drone-based photogrammetry, which is susceptible to environmental illumination variations and may lose depth information in deep pit regions, as well as MLS systems that often exhibit limited spatial resolution for localized defects, the proposed structured-light scanning framework provides a more stable depth-field reconstruction capability. This advantage becomes particularly evident in scenarios involving steep pit walls, complex textures, and severe geometric occlusion, where higher reconstruction robustness is required.

The proposed method is primarily intended for localized high-precision defect quantification rather than large-scale pavement network monitoring. Potential application scenarios include repair-material estimation, airport pavement inspection, racetrack maintenance, and localized engineering quality assessment. In these contexts, high geometric fidelity and accurate volumetric reconstruction are more critical than large-area acquisition efficiency.

**Figure 1:**
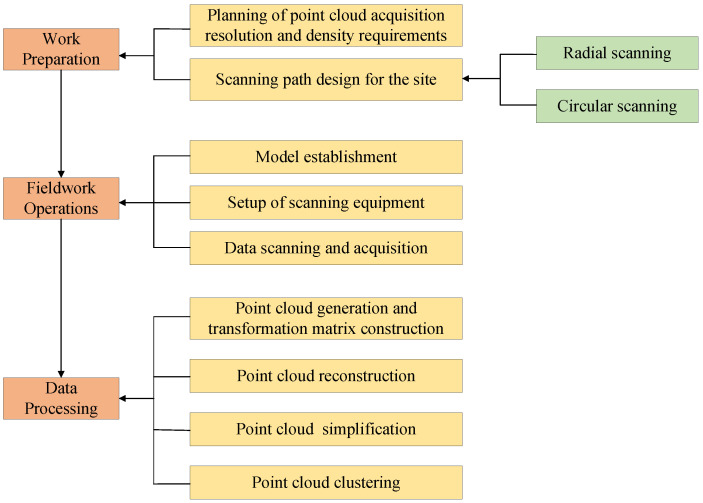
Flowchart of the integrated point cloud-based pit reconstruction and volume calculation system.

Figure 1 shows the main workflow diagram of this paper, which is primarily divided into three stages: planning and fieldwork, data preprocessing, and core calculation. First, the original point cloud of the test pit is obtained by planning the scanning strategy; subsequently, RANSAC plane fitting is utilized for pose normalization, and an adaptive simplification algorithm is combined to eliminate data redundancy while preserving morphological features; finally, through cubic polynomial piecewise fitting and the Simpson integration model, the automated and high-precision solution of the irregular pit volume is achieved.

## 3. Data Processing

### 3.1. Problem Description

Raw point cloud data acquired by a 3D laser scanner through circumferential scanning of pits is often massive [19]. If directly used in subsequent geometric fitting and volume calculation models, the data may significantly increase computational overhead, resulting in unnecessary processing costs and limiting the real-time performance required for on-site detection. Furthermore, due to the complex field operating environment, sensor limitations, and the rough reflective characteristics of the pit surface, the acquired data inevitably contains a considerable amount of interference noise. The presence of noise points and the effectiveness of their removal directly restrict the precision of subsequent cross-section extraction and volume quantification. The noise in the pit point cloud data collected by the 3D scanner circular scanning in the experiments consists of three parts: noise from equipment instability, viewpoint redundancy, and multipath reflection, as well as non-target point clouds from non-target interference [20].

To shorten the response time of the overall algorithm and eliminate the interference of noise on volume calculation, a systematic pretreatment process requires systematic preprocessing of the raw point clouds, including spatial pose correction, grid-based downsampling, and targeted morphological clustering simplification [21]. This aims to construct a concise and accurate underlying data foundation for subsequent high-precision volume calculation.

### 3.2. Pose Normalization

In this paper, when the 3D laser scanner collects pit point clouds, the acquired point clouds exhibit a non-horizontal tilted state due to the limitations of the equipment placement pose, making them unsuitable for direct use in subsequent geometric feature analysis and dimensional inspection. To address this issue, we propose a combined method of RANSAC plane fitting and rigid body transformation [22]. The bottom plane of the pit is first accurately fitted, and then the tilted bottom plane is corrected to a horizontal state through rigid body transformation, which ensures the integrity of the point cloud geometric features while meeting the pose requirements for industrial detection.

#### 3.2.1. Surface Reference Fitting Based on Ransac Algorithm

**Figure 2:**
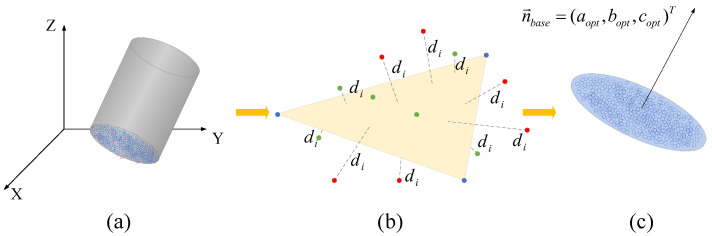
The RANSAC-based fitting process for the bottom plane of the pit. (**a**) Extraction of the bottom candidate point set, (**b**) Model verification and inlier identification, (**c**) Final result of the robustly fitted plane. The green dots represent the inliers (points belonging to the reference plane), while the red dots denote the outliers (environmental noise or non-planar points).

High-precision fitting of the bottom plane is a critical prerequisite for subsequent pose correction. To effectively mitigate the interference of noise and outliers such as convex hulls in 3D scanning point clouds, we implement the RANSAC algorithm to achieve robust fitting of the bottom plane. The core logic is based on random sampling, model verification, and the Boyer-Moore majority voting algorithm [23], with details in Algorithm 1. First, the algorithm filters the bottom candidate point set according to the coordinate axis distribution characteristics. Points in the raw point cloud whose Z-axis coordinates are within the 5% interval near the minimum value are selected as candidate samples as illustrated in Figure 2a. This sub-interval corresponds to the bottom end face of the pit and serves as the primary target area for plane fitting. Subsequently, employing a preset distance threshold, the algorithm cyclically executes random sampling, initial model construction, and inlier counting shown in Figure 2b. During each iteration, three non-collinear candidate points are randomly selected to determine the initial plane parameters, and the Euclidean distance [24] from all sample points to the model is calculated. Any points with a distance smaller than the preset threshold are identified as inliers and determined to be effective observations belonging to the bottom plane. After completing the preset number of optimization iterations, the plane parameters with the maximum inlier cardinality are selected as the final bottom plane model represented in Figure 2c.

As demonstrated in Algorithm 1, the core advantage of the RANSAC algorithm lies in its independence from predefined noise distributions. This algorithm effectively mitigates interference from outliers such as convex hulls and noise within the scanned point cloud by maximizing the proportion of inliers. Even when local data distortion exists in the raw point cloud, the system can still perform accurate discrimination based on the absolute distance tolerance defined in Algorithm 1. This process ensures the precise extraction of the true bottom plane model and the acquisition of the refined bottom point set $P_{ref}$.
**Algorithm 1** RANSAC-based reference plane estimation**Require:** Point cloud *P*, distance threshold ratio α, spatial filtering ratio β**Ensure:** Estimated plane model M, refined bottom points $P_{\mathrm{ref}}$, status flag *S* 1:Find $z_{\min}$ as the minimum elevation value in the point cloud *P* 2:Compute the global elevation span $\Delta z=\max\left(P_{z}\right)-z_{\min}$ 3:Determine the spatial filtering threshold $z_{th}=z_{\min}+\Delta z\cdot \beta$ 4:**for** each sampling point $p_{i}\in P$ **do** 5:    **if** $p_{i,z}<z_{th}$ **then** 6:        Insert $p_{i}$ into candidate subset $P_{\mathrm{cand}}$ 7:    **end if** 8:**end for** 9:**if** the cardinality of $P_{\mathrm{cand}}<3$ **then**10:    S←False11:    **return** $⌀,P_{\mathrm{cand}},S$12:**end if**13:Calculate the absolute distance tolerance $d_{\max}=\alpha \cdot \left(\max\left(P\right)-\min\left(P\right)\right)$14:Execute RANSAC fitting on $P_{\mathrm{cand}}$ to estimate plane parameters M15:**if**M is successfully converged **then**16:    S←True17:    **for** each $p_{j}\in P$ **do**18:        **if** distance $\left(p_{j},M\right)\leq d_{\max}$ **then**19:           Insert $p_{j}$ into $P_{\mathrm{ref}}$20:        **end if**21:    **end for**22:**else**23:    S←False24:    $P_{\mathrm{ref}}\leftarrow P_{\mathrm{cand}}$25:**end if**26:**return**$M,P_{\mathrm{ref}}$ and *S*

#### 3.2.2. Rigid Body Transformation

The rotation matrix is constructed using the normal vector of the bottom plane obtained via RANSAC fitting as the geometric reference, aiming to achieve a linear mapping of the normal vector toward the Z-axis direction. The specific calculation process is completed with the aid of Rodrigues’ Rotation Formula. By calculating the cross product of the normal vector of the bottom plane and the Z-axis unit vector, the rotation axis is determined, and the dot product of the two is used to solve for the rotation angle, thereby constructing an orthogonal transformation matrix capable of accurately redirecting the bottom normal vector to the Z-axis [25]. For special working conditions where the normal vector and the Z-axis are distributed in completely opposite directions, the algorithm will directly invoke a 180° rotation matrix to perform pose correction.

After obtaining the unit normal vector $n=\left(n_{x},n_{y},n_{z}\right)$ of the pit bottom reference plane through the RANSAC algorithm, it is necessary to construct a rigid body transformation matrix to correct this normal vector to the standard Z-axis direction k. This study utilizes Rodrigues’ Rotation Formula to implement this linear mapping process. First, the cross product of the initial normal vector n and the target vector k is calculated to determine the rotation axis *v*, as in (1), and the dot product of the two is calculated to obtain the cosine value *c* of the rotation angle θ, as in (2).

(1) $$v=n\times k=\left(n_{y},-n_{x},0\right)$$

(2) $$c=n\cdot k=n_{z}$$

Let the skew-symmetric matrix of the rotation axis v be V, as in (3).

(3) $$V=\left[\begin{array}{ccc} 0 & 0 & -n_{x}\\ 0 & 0 & -n_{y}\\ n_{x} & n_{y} & 0 \end{array}\right]$$

According to Rodrigues’ formula, the analytical expression of the rotation matrix R is given by (4).

(4) $$R=I+V+V^{2}\frac{1-c}{1-c^{2}}$$

where I is the identity matrix. For extreme working conditions where the normal vector is completely opposite to the Z-axis direction, the algorithm will directly invoke a specific flipping matrix to perform pose correction. Finally, by coupling the rotation matrix R with the translation vector T, a global transformation operator is constructed to reposition the raw point cloud $P_{original}$, as in (5).

(5) $$P_{aligned}=R\cdot P_{original}+T$$

**Figure 3:**
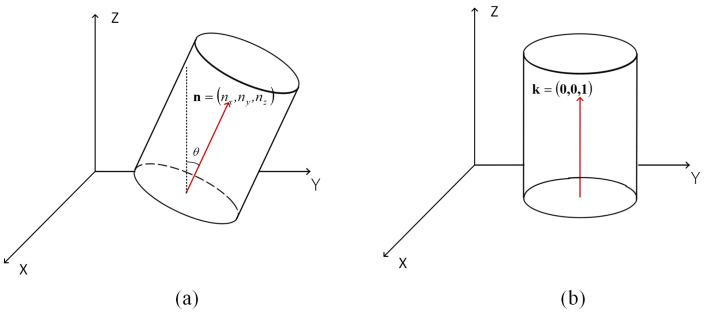
Comparison of pit point cloud pose correction. (**a**) Schematic diagram of original point cloud posture. (**b**) Schematic diagram of point cloud posture after posture correction.

This transformation process possesses orthogonality constraints, which can ensure that the corrected pit top surface is strictly at the horizontal reference position while keeping the original geometric spacing of the point cloud unchanged, thereby eliminating systematic tilt deviations for subsequent cross-section extraction [26] as illustrated in Figure 3.

### 3.3. Planar Geometric Reconstruction

To address the incomplete topological structure caused by the missing top surface of the pit point clouds, this paper proposes a geometric reconstruction method based on segmented fitting of edge points, aiming to achieve the complete completion of the pit structure’s top surface [27]. The method begins by extracting the sampling points in the top 20% of the Z-axis range from the point cloud data as the upper edge candidate points, where the edge point sequence is divided into several continuous sub-intervals by X-coordinates using sorting and segmentation strategies. For the edge points within each sub-interval, the local edge lines are fitted by filtering the extreme points of Y-coordinates combined with the geometric constraints of two-point collinearity. This linear envelope then serves as the basis for an interpolation algorithm executed within the interval to generate high-density top surface feature points, leading to the final topological restoration of the pit structure’s top surface by fusing the synthetic point clouds with the raw observation data.

The superiority of this method lies in the utilization of segmented fitting and extreme point geometric constraints, which effectively adapts to the edge features of quasi-cylindrical structures. Since the upper edge of the pit is distributed in a circular pattern in space, the non-linear circular trajectory can be discretized into several local linear segments through X-coordinate segmentation, while the extreme points of Y-coordinates correspond to the radial antipodal points of the circular edge. The linear model established in this way can accurately represent the edge orientation of the segment. The top surface point clouds generated using linear interpolation are distributed along the edge trajectory, ensuring the geometric continuity [28] between the reconstructed top surface and the original pit structure.

In the top surface fitting process, the generation of a single-segment edge line follows the linear interpolation criterion. For the extreme points of Y-coordinates $P_{1}\left(x_{1},y_{1},z_{1}\right)$ and $P_{2}\left(x_{2},y_{2},z_{2}\right)$ within the interval, the coordinates of their interpolation points are calculated as in (6).

(6) $$\left\{\begin{array}{c} x=x_{1}+t\left(x_{2}-x_{1}\right)\\ y=y_{1}+t\left(y_{2}-y_{1}\right)\\ z=z_{1}+t\left(z_{2}-z_{1}\right) \end{array}\right.,t\in \left[0,1\right]$$

where *t* is the interpolation parameter, and the density of *t* values is controlled by setting a thinning factor. The magnitude of the thinning factor is positively correlated with the sampling interval of *t*; that is, the larger the thinning factor, the fewer the number of points generated. Finally, the interpolation points generated by each segment are integrated to form a complete point cloud top surface with geometric consistency.

### 3.4. Data Gridding

In the process of feature analysis and geometric reconstruction of pit point clouds, the Z-axis coordinates of the raw observation data exhibit piecewise-continuous distribution characteristics. Limited by the precision of 3D scanning equipment, the Z-coordinates of adjacent points often have slight numerical fluctuations. This irregular numerical discreteness significantly increases the complexity of subsequent contour extraction and calculation of cross-sectional dimensions. To this end, this paper performs layered gridding in the Z-axis direction on the corrected pit point clouds. Its core objective is to merge the continuously distributed height information into discrete height layers at preset intervals, thereby constructing equidistant Z-axis structured point clouds. This pretreatment method achieves significant data compression while preserving key geometric features, providing a unified height datum for subsequent layered extraction of cross-sectional contours and analysis of radial dimension evolution [29].

The theoretical logic of layered gridding for point cloud Z-coordinates is based on numerical merging and spatial discretization representation, and the specific technical path is based on the equidistant quantization criterion. The algorithm extracts the global Z-axis range of the corrected point cloud and clarifies the quantization interval by determining the extreme values of the Z-coordinates. By setting a fixed height layer interval z, the original coordinate value of each point is mapped according to the rule of rounding to the nearest integer multiple of z. This standardization of the Z-coordinate is completed as in (7).

(7) $$Z_{norm}=z_{Interval}\cdot round\left(\frac{Z_{raw}}{z_{Interval}}\right)$$

The above quantization process essentially divides the continuous Z-axis space into equidistant gridding layers, making the Z-coordinate values of all points within the same gridding layer completely consistent, thereby achieving structured layering of the point cloud in the axial direction.

**Figure 4:**
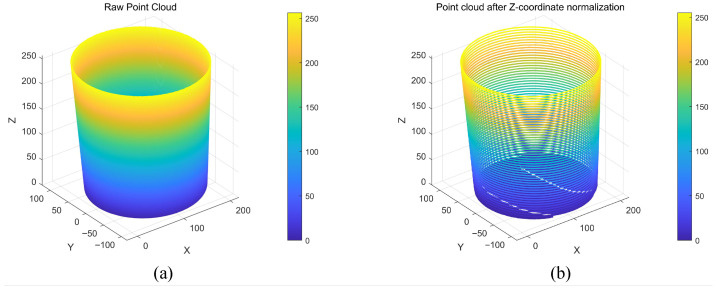
Visual comparison of point cloud gridding results. (**a**) raw point cloud with continuous axial distribution, (**b**) processed point cloud with equidistant discoid stratification.

The advantage of this processing method is that it only performs discretized merging on the Z-axis without changing the spatial distribution of the point cloud in the X-Y plane, which preserves the integrity of geometric features of the barrel-shaped part in the radial direction and constructs a regular height layering system. The comparison effect after gridding processing is shown in Figure 4. Each discrete height layer corresponds to an axial cross-section of the barrel-shaped part, which can be directly used to extract cross-sectional contours and calculate radial dimensions, effectively eliminating the computational complexity caused by the discreteness of the raw data [30]. Meanwhile, by counting the distribution density of the quantized discrete Z-coordinates, the axial height features of the pits can be intuitively grasped, providing a clear layering basis for subsequent dimensional inspection tasks.

### 3.5. Point Cloud Thinning

After pretreatment such as 3D scanning, top surface reconstruction, and Z-axis gridding, the point clouds of each height layer still exhibit significant data redundancy, non-uniform local density, and a small amount of stray noise. If the raw point clouds are directly utilized for cross-sectional contour extraction and volume calculation, it will lead to a substantial decrease in processing efficiency and compromised calculation accuracy. To this end, this paper proposes a point cloud simplification strategy with layered topology preservation and global integration. This strategy aims to achieve point cloud denoising and data compression through the combined use of thinning and clustering algorithms, provided that the axial layered topological structure and radial geometric feature integrity of the pit are ensured [31]. In the specific implementation flow, the algorithm first completes adaptive thinning of the point clouds in each height layer based on local density features, followed by the merging of redundant observation points through point cloud clustering and eigenvalue aggregation. This processing approach can significantly reduce the point clouds scale while ensuring the closure and accuracy of the cross-sectional contours at each height layer, providing a high-quality data foundation for the precise calculation of cross-sectional areas and total volume solution in subsequent sections. Since the distribution density of pit point clouds varies significantly across different height layers, a uniform thinning method with a fixed step size struggles to balance the relationship between redundancy removal and feature preservation. In view of this phenomenon, this paper further constructs a three-level thinning system consisting of local density estimation, gradient adaptive spacing, and local grid optimal sampling [32]. The core logic of this system lies in dynamically adjusting sampling weights according to the local spatial distribution features of the point cloud, thereby achieving effective screening for high-density areas and refined maintenance of low-density key geometric features.

#### 3.5.1. Local Density Estimation and Adaptive Sampling

To address the issue of non-uniform density distribution in each height layer of the pit point cloud, this paper first executes a local density estimation algorithm based on K-NN search. This algorithm quantitatively characterizes the local thinning of the point cloud by counting the spatial distribution relationship between the sampling point and its neighborhood point set, providing data support for the subsequent setting of adaptive sampling spacing.

For any sampling point $p_{i}\left(x_{i},y_{i},z_{i}\right)$ within the height layer, K-nearest neighbor (K-NN) search algorithm is used to retrieve its *k* nearest neighbor points in 3D Euclidean space, and the average Euclidean distance $\bar{d_{i}}$ from this point to its neighborhood point set is calculated, serving as a micro-geometric operator to measure the local point cloud discreteness as in (8).

(8) $$\bar{d_{i}}=\frac{1}{k}\sum_{j=1}^{k}\sqrt{{\left(x_{i}-x_{j}\right)}^{2}+{\left(y_{i}-y_{j}\right)}^{2}+{\left(z_{i}-z_{j}\right)}^{2}}$$

On this basis, the local density $\rho_{i}$ is defined as the reciprocal of the average distance, which is used to quantitatively describe the aggregation state and geometric feature abundance around the sampling point, as expressed in (9).

(9) $$\rho_{i}=\frac{1}{\bar{d_{i}}}$$

The physical logic of the above definition lies in constructing a dynamic feedback mechanism. When $\bar{d_{i}}$ tends toward a smaller value, it reflects that the sampling points are highly aggregated within the current local neighborhood, corresponding to the redundant data-intensive area of the pit model. At this time, the value of $\rho_{i}$ increases accordingly, based on which the algorithm determines that this area possesses high simplification potential, requiring the removal of excess observational redundancy by increasing the subsequent sampling step size. Conversely, if $\bar{d_{i}}$ is large and $\rho_{i}$ is small, it indicates that the point cloud distribution in this area is sparse, mostly corresponding to the feature transition points or scanning blind zone edges of the pit, necessitating strict limits on the simplification intensity to maintain the geometric integrity of the original structure [33]. This adaptive adjustment mechanism based on local density feedback effectively overcomes the limitations of traditional uniform thinning algorithms for complex pit morphologies, ensuring that the simplified point cloud retains high-fidelity representation of radial dimensional variation while significantly reducing data volume.

#### 3.5.2. Construction of a Gradient Adaptive Sampling Interval Mapping Model

After acquiring the local density distribution features of the point clouds in each height layer, to achieve differentiated data compression, this paper constructs a gradient adaptive sampling interval mapping model based on the extreme range of local density. The core objective of this model is to map the continuously varying density index $\rho_{i}$ to a spatial sampling step size $s_{i}$, thereby employing fine-grained sampling in low-density areas with complex geometric structures, and executing coarse-grained screening in high-density areas with data redundancy [34].

The specific mapping logic involves analyzing the global distribution of point cloud density within the current processing layer to extract the maximum density $\rho_{\max}$ and minimum density $\rho_{\min}$. Given a base sampling interval $s_{0}$, the adaptive sampling interval *s* corresponding to the sampling point $p_{i}$ is calculated based on the linear interpolation principle, as in (10).

(10) $$s_{i}=0.5s_{0}+\frac{\rho_{i}-\rho_{\min}}{\rho_{\max}-\rho_{\min}}\times 1.5s_{0}$$

To prevent topological distortion of local features or under-sampling of the pit caused by extreme interval settings, this paper strictly constrains the sampling step size $s_{i}$ within the interval $\left[0.5s_{0},2s_{0}\right]$. Through this gradient adjustment mechanism, the algorithm can implement large-interval sampling in high-density areas to improve computational efficiency, while executing small-interval sampling in low-density areas to enhance feature preservation.

This gradient mapping method ensures a dynamic equilibrium between sampling intervals and local geometric complexity. By applying non-linear constraints to the sampling intervals, the issue of insufficient robustness exhibited by fixed step sizes when processing different parts of the pit components is effectively resolved. The generated variable-interval sampling point set not only reduces the redundancy of the raw data but also provides a point cloud input with a more reasonable spatial distribution for the subsequent closed fitting of cross-sectional contours.

#### 3.5.3. Local Grid Optimal Sampling Strategy

After obtaining the adaptive sampling interval $s_{i}$ for each sampling point, this paper adopts a local grid optimal sampling strategy to execute point cloud thinning [35]. This step aims to maximize the preservation of the original geometric position features and cross-sectional topological structure of the pit while eliminating redundant data through refined spatial partitioning and centroid constraints.

The implementation begins by constructing a 3D local grid with a side length of $s_{i}$, centered at the current sampling point c. The spatial boundary of this grid is defined as in (11).

(11) $$\left\{\begin{array}{c} x\in \left[x_{i}-\frac{s_{i}}{2},x_{i}+\frac{s_{i}}{2}\right]\\ y\in \left[y_{i}-\frac{s_{i}}{2},y_{i}+\frac{s_{i}}{2}\right]\\ z\in \left[z_{i}-\frac{s_{i}}{2},z_{i}+\frac{s_{i}}{2}\right] \end{array}\right.$$

Subsequently, the algorithm retrieves and locks all candidate point clouds falling within the grid bounding box, and calculates the 3D Euclidean distance $d_{c}$ from each candidate point $p_{i}$ to the grid center *p*, as expressed in (12).

(12) $$d_{c}=\sqrt{{\left(x-x_{i}\right)}^{2}+{\left(y-y_{i}\right)}^{2}+{\left(z-z_{i}\right)}^{2}}$$

On this basis, the point with the minimum $d_{c}$ value is selected as the optimal sampling point for this local area, and the remaining points within the grid are simultaneously marked as sampled status to avoid computational redundancy caused by repeated selection. Through grid partitioning and dynamic selection of optimal sampling points, the spatial uniformity of the simplified point cloud is preserved while maintaining high-precision positional information at the local scale. Ultimately, this strategy achieves adaptive thinning of the point clouds in each height layer, ensuring the integrity of the cross-sectional topological structure of the pits while eliminating stray noise and redundant points.

**Figure 5:**
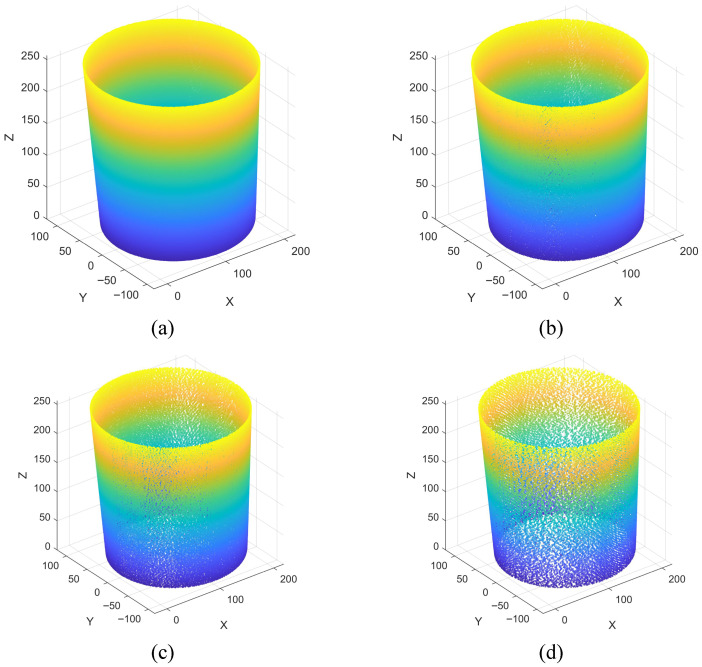
Reconstruction models under different sparsity densities: (**a**) Original point cloud, (**b**) Thinned with $s_{0}$ = 3, (**c**) Thinned with $s_{0}$ = 7, showing the most appropriate density, (**d**) Thinned with $s_{0}$ = 14, resulting in excessive sparsity. The color represents the height (Z-coordinate) of the point cloud.

The distribution effect of the processed point cloud is shown in Figure 5: by comparing the reconstruction models under different thinning densities, it can be found that the method in this paper, on the basis of significantly reducing the data volume, can still clearly outline the subtle morphological features of the pit wall surface, laying a solid data foundation for the subsequent precise fitting of cross-sectional areas.

### 3.6. Point Cloud Clustering

For the layered point clouds of barrel-shaped parts after simplification processing, it is still necessary to carry out refined screening and aggregation according to the morphological features of different cross-sections [36]. This paper constructs an aggregation strategy based on morphological feature quantization, polar coordinate constraints, and banded interval screening, aiming to eliminate stray points inside and outside the contour while preserving core features.

The core theoretical basis of this method is the quantitative discrimination of geometric morphology and polar coordinate space constraints. For the XY plane coordinates of a single-layer point cloud, basic geometric features are calculated to discriminate the cross-sectional morphology. The algorithm first performs geometric discrimination on the single-layer point cloud. By extracting dimensional indicators such as the extreme range difference ${∆}_{range}$ of the XY plane coordinates and the cross-dimensional differences ∆ $y_{x}$ and ∆ $x_{y}$, where ∆ $y_{x}$ denotes the Y range at the maximum X and ∆ $x_{y}$ denotes the X range at the maximum Y, a morphological determination criterion is established: if any of the conditions ${∆}_{range}>50\;\mathrm{mm}$, ∆ $y_{x}>80\;\mathrm{mm}$, or ∆ $x_{y}>80\;\mathrm{mm}$ is satisfied, the current cross-section is determined to be crescent-shaped; otherwise, it is determined to be near-circular.

For near-circular cross-sections, an aggregation strategy using circle center and radius constraints is adopted. Taking the XY range center $O\left(\bar{x},\bar{y}\right)$ as the fitted circle center, the Euclidean distance $d_{i}$ from each sampling point to the circle center is calculated as in (13).

(13) $$d_{i}=\sqrt{{\left(x_{i}-\bar{x}\right)}^{2}+{\left(y_{i}-\bar{y}\right)}^{2}}$$

Select the maximum distance as the reference radius $r_{base}=\max\left(d_{i}\right)$, and screen the points within the banded interval $\left[r_{base}-20,r_{base}+20\right]$ as core contour points, thereby eliminating noise near the center of the circle and points excessively deviating from the contour.

For crescent-shaped cross-sections, a polar coordinate space constraint strategy is adopted. First, determine the center of the dense crescent area $O_{d}\left({\bar{x}}_{d},{\bar{y}}_{d}\right)$, and calculate the polar angle $\theta_{i}$ and polar radius $d_{di}$ of each point relative to this center, as in (14) and (15).

(14) $$\theta_{i}=\arctan2\left(y_{i}-{\bar{y}}_{d},x_{i}-{\bar{x}}_{d}\right)$$

(15) $$d_{di}=\sqrt{{\left(x_{i}-{\bar{x}}_{d}\right)}^{2}+{\left(y_{i}-{\bar{y}}_{d}\right)}^{2}}$$

**Figure 6:**
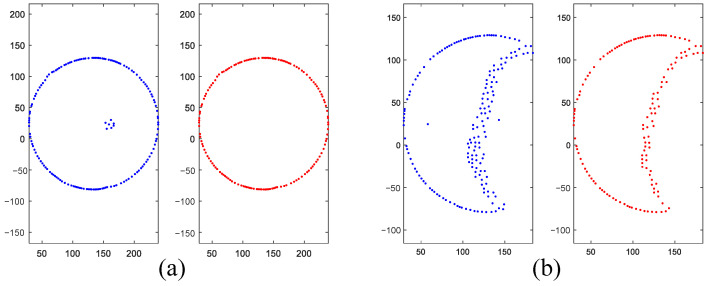
Differentiated point cloud aggregation for various cross-sectional morphologies. (**a**) near-circular slice aggregation. (**b**) crescent-shaped slice aggregation. The red dots represent the point cloud distribution before aggregation, and the blue dots represent the result after aggregation.

Within the limited polar angle range $\left(-\frac{\pi }{2},\pi \right)$, the polar radius are sorted to extract the 10% quantile $d_{low}$ and the 90% quantile $d_{up}$, and the constraint interval is relaxed to $\left[d_{low}-10,d_{up}+10\right]$. Finally, points that simultaneously satisfy the polar angle and polar radius constraints are retained as the core feature points of the crescent-shaped cross-section. Through the aforementioned differentiated aggregation strategy based on morphological features, this paper achieves the precise purification of point clouds for different cross-section types as shown in Figure 6. The near-circular cross-sections rely on circle center and radius constraints to ensure the circularity integrity of the contour, while the crescent-shaped cross-sections rely on angle and distance constraints under the polar coordinate system to preserve arc trajectory features, ultimately fully retaining the core geometric features of the pit cross-sections at each height layer [37].

## 4. Volume Calculation

### 4.1. Cross-Sectional Area Calculation

Cross-sectional area calculation is the core step for pit volume measurement, and its precision directly determines the reliability of the final result [38]. Aiming at the Preprocessed point clouds of each height layer after pretreatment, this paper adopts a method combining partitioned piecewise polynomial fitting with integral solution, constructing an area calculation method suitable for irregular cross-sections. This method achieves the quantitative characterization of the cross-sectional area of each layer by accurately fitting the contour curves of the discrete point clouds and performing analytical integration. The core theoretical basis of this method is the polynomial curve fitting principle and the definite integral area solution concept [39]. Polynomial fitting relies on the least squares criterion, which constructs appropriate-order polynomial functions to approximate discrete point cloud contours—effectively smoothing residual noise and preserving contour geometric feature [8]. Considering the irregularity of the pit cross-sectional contours in radial distribution, it is difficult for a single polynomial function to maintain high goodness of fit across the entire range. Therefore, this paper adopts a partitioning strategy to first perform interval division on the single-layer point cloud, laying a foundation for the subsequent piecewise precise fitting.

#### 4.1.1. Cubic Polynomial Fitting and Normal Equation Solving

In view of the discrete characteristics of single-layer point clouds, this paper chooses to use cubic polynomials for curve fitting. Cubic polynomials possess excellent adaptability to curvature changes and are capable of accurately capturing the arc-shaped features of the contour while avoiding curve distortion caused by overfitting of high-order polynomials [40]. Through the least squares algorithm, the coefficients corresponding to each segment of the point cloud are solved to minimize the sum of squared residuals between the polynomial curve and the discrete point cloud, ensuring that the fitted curve can approximate the actual contour to the greatest extent.

After determining the use of piecewise-cubic polynomials as the fitting primitives, for any set of discrete points $\left(x_{i},y_{i}\right)\left(i=1,2,\ldots ,n\right)$ in a segment of the single-layer point cloud, the fitting goal is to construct a cubic polynomial function as in (16).

(16) $$f\left(x\right)=a_{0}+a_{1}x+a_{2}x^{2}+a_{3}x^{3}$$

To minimize the sum of squared residuals *E* between the fitted curve and the discrete observations, the least squares criterion must be satisfied as expressed in (17).

(17) $$\begin{array}{cc} E(a_{0},a_{1},a_{2},a_{3}) & =\sum_{i=1}^{n}[y_{i}-(a_{0}+a_{1}x_{i}\\ \; & \;+a_{2}x_{i}^{2}+a_{3}x_{i}^{3}{)]}^{2}\to \min \end{array}$$

By taking partial derivatives of the residual function *E* with respect to each undetermined coefficient $a_{j}$ and setting them to zero, the nonlinear approximation problem can be transformed into a problem of solving linear equations. The normal equations are constructed as in (18).

(18) $$X^{T}\mathrm{Xa}=X^{T}y$$

where X is a Vandermonde-type design matrix, $a={\left[a_{0},a_{1},a_{2},a_{3}\right]}^{T}$ is the coefficient vector, and $y={\left[y_{1},y_{2},\ldots ,y_{n}\right]}^{T}$ is the observation vector. The form of the design matrix X is expressed as in (19).

(19) $$X=\left[\begin{array}{cccc} 1 & x_{1} & x_{1}^{2} & x_{1}^{3}\\ 1 & x_{2} & x_{2}^{2} & x_{2}^{3}\\ \vdots & \vdots & \vdots & \vdots \\ 1 & x_{n} & x_{n}^{2} & x_{n}^{3} \end{array}\right]$$

$a={\left(X^{T}X\right)}^{-1}X^{T}y$ can be solved, thereby uniquely determining the fitting polynomial $f\left(x\right)$ within this piecewise interval.

#### 4.1.2. Partitioned Area Solution Based on Definite Integral

Considering the possible asymmetry of the barrel-shaped part cross-sectional contour (such as Y-direction offset caused by local convex hulls), this paper introduces a calculation method of upper and lower half-plane partitioning. The partition reference value $y_{mid}$ is calculated based on the extreme values of the single-layer point cloud Y-coordinates, as in (20).

(20) $$y_{mid}=\frac{1}{2}\left(\max\left(y_{i}\right)+\min\left(y_{i}\right)\right)$$

**Figure 7:**
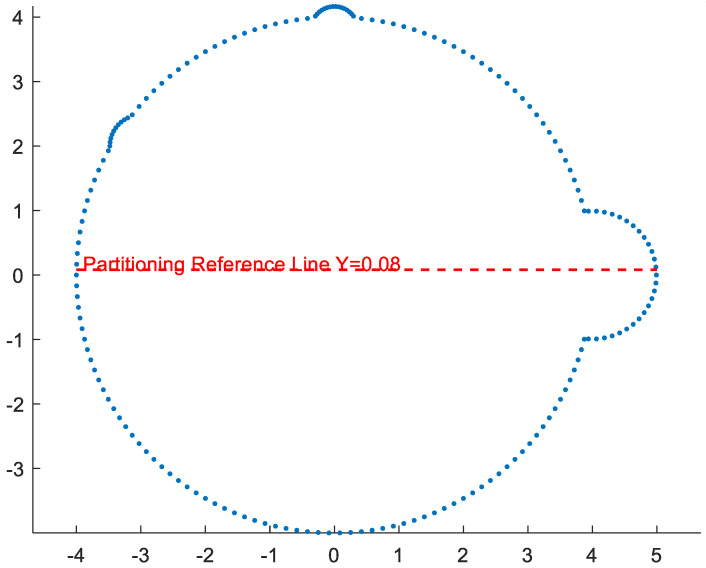
Partitioning of the cross-sectional point cloud into upper and lower half-planes.

Using this reference, the point cloud is divided into an upper half-plane and a lower half-plane, as shown in Figure 7. After partitioning, the point cloud is sorted in ascending order according to the X-axis coordinates to ensure the continuity of the point sequence during the fitting process and avoid the curve intersection phenomenon. For any cubic curve segment obtained by fitting, if its projection interval on the X-axis is $\left[x_{\min},x_{\max}\right]\left(x_{\min}=\min\left(x_{i}\right),x_{\max}=\max\left(x_{i}\right)\right)$, the area $S_{seg}$ enclosed by this curve segment and the X-axis can be obtained via definite integration expressed in (21).

(21) $$S_{seg}=\int_{x_{\min}}^{x_{\max}}\left(a_{0}+a_{1}x+a_{2}x^{2}+a_{3}x^{3}\right)dx$$

**Figure 8:**
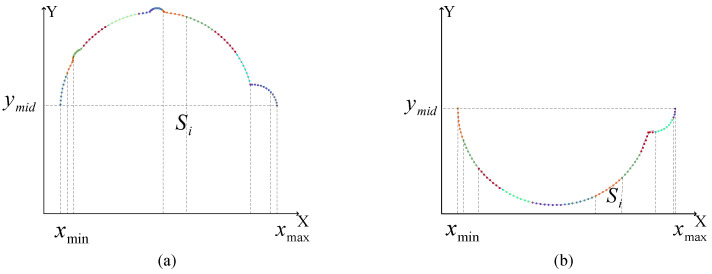
Geometric representation of the piecewise integration process for area calculation. (**a**) Piecewise integration layout of the upper half-plane for calculating $S_{up}$, (**b**) Piecewise integration layout of the lower half-plane for calculating $S_{low}$.

The sum of all piecewise areas in the upper half-plane is accumulated to obtain $S_{up}$ and the sum of areas in the lower half-plane is accumulated to obtain $S_{low}$. The integration process for these two regions is visualized in Figure 8. Since the cross-sectional contour is jointly enclosed by the upper and lower curves, the final single-layer cross-sectional area $S_{total}$ is the absolute value of the difference between the two, as in (22).

(22) $$S_{total}=\left|S_{up}-S_{low}\right|$$

This difference calculation method effectively eliminates the area deviation caused by coordinate axis projection and can accurately reflect the actual physical area of irregular cross-sections. Verification against simulated point clouds generated from standard shapes shows that the relative error of this polynomial fitting integration method is less than 0.1%. Compared with the traditional trapezoidal integration method, the method in this paper can effectively filter out residual point cloud noise through the smoothing characteristics of polynomials, maintaining high solution precision even when point cloud density is low, providing core technical support for the high-precision solution of pit volume.

### 4.2. Cross-Sectional Area Data Processing

To address issues like negative areas and outliers from abnormal discrete point distribution in layered cross-sectional area results, this paper proposes a dynamic outlier removal method combining MAD and piecewise sliding windows [41]. The core of this method relies on the robust statistical characteristics of MAD to construct dynamic threshold intervals, combined with sliding window analysis to capture the local distribution features of data, and set differentiated parameters by adapting to the phased pattern of cross-sectional data—steady in the early stage and gradual in the later stage—to achieve precise identification and removal of outliers [42].

Robust statistical principle of MAD: The key to outlier discrimination accuracy lies in selecting a dispersion index that is insensitive to extreme values. Compared with the standard deviation, which is easily disturbed by extreme values, MAD, as a robust statistic, can effectively reflect the true degree of dispersion of data around the median. For the area data set $W_{i}$ within the sliding window corresponding to the *i*-th data point, the MAD calculation formula is as follows calculated as in (23).

(23) $$MAD\left(W_{i}\right)=\mathrm{median}\left(\left|x_{j}-\mathrm{median}\left(W_{i}\right)\right|\right)$$

where $x_{j}$ is the *j*-th data point within the window, and $median\left(\cdot \right)$ is the median solution operator. The median solution characteristic ensures that MAD is not affected by individual extreme values within the window; even in the presence of outlying abnormal area values, it can still accurately represent the central tendency of local data, providing a reliable dispersion benchmark for anomaly determination. The dynamic anomaly determination threshold interval constructed based on MAD is expressed as in (24).

(24) $$\begin{array}{cc} {}[ & median\left(W_{i}\right)-k\cdot MAD\left(W_{i}\right),\\ \; & median\left(W_{i}\right)+k\cdot MAD\left(W_{i}\right)] \end{array}$$

In (24), *k* is the MAD magnification factor, used to regulate the threshold interval width. If measurement point falls outside this interval range, it is determined to be a non-physical outlier and is subsequently removed.

The core of sliding window analysis lies in reflecting the contextual distribution pattern of data points through the statistical features of local data subsets. Aiming at the distribution characteristics of pit cross-sectional areas, which are characterized by stability in the early stage and gradual changes in the later stage, we design a piecewise sliding window adaptation strategy.

- Window Size Adaptation: The early stable segment adopts a wide window of 10 data points, utilizing more local data to smooth random fluctuations and ensure the stability of the threshold interval; the later gradual segment adopts a narrow window of 6 data points to enhance the response sensitivity of the window to data gradient trends, avoiding the masking of true gradient features due to excessively wide windows.
- Boundary window compensation: Aiming at the window boundary effect of the start and end data points, a window is constructed for the first 3 data points using a subsequent fixed-length local data set, and for the last 3 data points using a preceding fixed-length local data set, ensuring that the threshold interval of the boundary data can still reflect the local data distribution characteristics, avoiding the misjudgment of valid boundary data as outliers.
- Piecewise benchmark division: Taking the height value of $z_{max}-30\;\mathrm{mm}$ as the automatic boundary point, the data is divided into an early stable segment and a later gradual segment, with differentiated MAD magnification factors *k* set respectively. The early high factor *k* adapts to the low dispersion characteristics of the stable segment, avoiding the erroneous removal of valid data; the later low factor adapts to the dynamic change characteristics of the gradual segment, accurately identifying outliers that deviate from the gradient trend.

**Figure 9:**
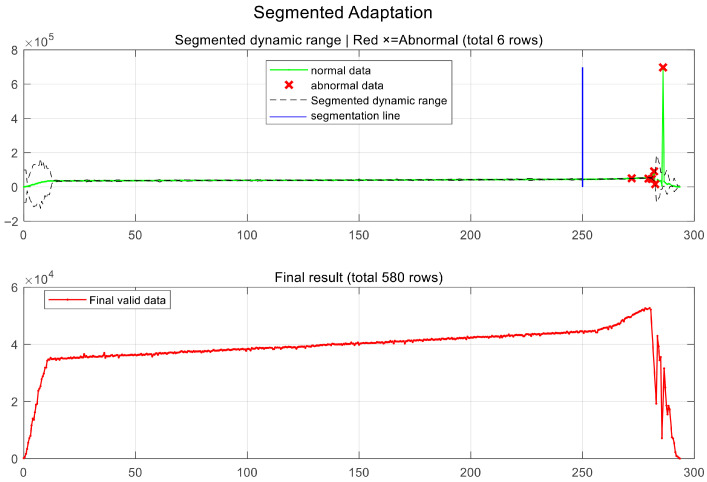
Visualization of the outlier removal process based on the piecewise MAD sliding window algorithm.

Through the MAD dynamic rejection logic based on a segmented sliding window shown in Algorithm 2, this study achieves refined noise reduction for the cross-sectional area sequence. To further verify the effectiveness of the proposed algorithm, noise reduction was performed on the acquired pit cross-sectional area sequence. The segmented adaptation performance is illustrated in Figure 9.
**Algorithm 2** Piecewise sliding window dynamic outlier removal based on MAD**Require:** Raw cross-sectional area dataset $D={\left\{\left(x_{i},y_{i}\right)\right\}}_{i=1}^{n}$; parameters for stable stage $\left\{W_{pre},k_{pre}\right\}$ and transition stage $\left\{W_{post},k_{post}\right\}$; segmentation height threshold $H_{split}$**Ensure:** Refined dataset $D_{final}$ after outlier removal 1:**for** each $d_{i}\in D$ **do** 2:    **if** $x_{i}<0$ **then** 3:        Remove $d_{i}$ from *D* 4:    **end if** 5:**end for** 6:Find the split index *m* where $y_{m}\geq H_{split}$ 7:**for** each $i\in [1,n^{\prime }]$ **do** 8:    **if** i≤m **then** 9:        $\left\{W,k\right\}\leftarrow \left\{W_{pre},k_{pre}\right\}$10:    **else**11:        $\left\{W,k\right\}\leftarrow \left\{W_{post},k_{post}\right\}$12:    **end if**13:    **if** i≤3 **then**14:        win_range←[1,W]15:    **else if** $i\geq n^{\prime }-2$ **then**16:        $win\_range\leftarrow [n^{\prime }-W+1,n^{\prime }]$17:    **else**18:        win_range←[i−W+1,i]19:    **end if**20:    Calculate $\mathrm{median}(W_{i})$ and $\mathrm{MAD}(W_{i})$ for current window21:    $\left[L_{i},U_{i}\right]\leftarrow \left[\mathrm{median}\left(W_{i}\right)-k\cdot \mathrm{MAD}\left(W_{i}\right),\mathrm{median}\left(W_{i}\right)+k\cdot \mathrm{MAD}\left(W_{i}\right)\right]$22:    **if** $x_{i}<L_{i}$ **or** $x_{i}>U_{i}$ **then**23:        Mark $d_{i}$ as abnormal24:    **end if**25:**end for**26:**return** $D_{final}=\left\{d_{i}\in D|d_{i}\;\mathrm{is}\;\mathrm{not}\;\mathrm{abnormal}\right\}$

The upper portion displays the dynamic threshold envelope as it evolves with the area data. It can be observed that the algorithm maintains a narrow discrimination interval during the early stable phase, while automatically adjusting its sensitivity during the later transitional phase. This enables the successful identification and elimination of abnormal peaks caused by discrete point cloud interference marked with red crosses. The lower portion presents the area sequence after the removal of outliers, which accurately reconstructs the geometric evolution of the pit inner wall from a gradual slope to the rapid contraction at the bottom.

### 4.3. Integral Volume Calculation Based on Slicing

Based on the precise solution results of the cross-sectional area of each height layer, this paper combines Simpson’s rule and the frustum volume formula to construct a volume calculation method suitable for irregular pits. This method makes full use of the height discretization features of the layered point clouds. It uses different calculation strategies according to the parity of the total layer count, which not only ensures the integration accuracy of the regular layered areas but also adapts to the geometric characteristics of the terminal layers, achieving a high-precision solution for the overall volume [43].

The core theoretical basis of volume calculation is the numerical integration principle in calculus and the frustum volume formula in solid geometry. Simpson’s rule, as a second-order Newton-Cotes formula, has the core idea of dividing the integration interval into several even segments and approximating the integrand function with quadratic parabolas. Compared with the trapezoidal integration method, it possesses higher calculation accuracy and is suitable for regular scenarios where the number of layers is odd. For the cross-sectional area sequence discretely distributed along the Z-axis direction, assuming the heights of three adjacent layers are $z_{i},z_{i+1},z_{i+2}$, and the corresponding cross-sectional areas are $S_{i},S_{i+1},S_{i+2}$, where the layer heights satisfy the strictly increasing characteristic of equal or non-equal spacing, the volume infinitesimal formula constituted by these three layers calculated by Simpson’s rule is calculated as in (25).

(25) $$V_{i}=\frac{h_{i}}{6}\left(S_{i}+4S_{i+1}+S_{i+2}\right)$$

where $h_{i}=z_{i+2}-z_{i}$ is the total height of these three layers. This formula integrates the contributions of three cross-sectional areas through weighted averaging, which can accurately reflect the nonlinear characteristics of cross-sectional area variations with height.

**Figure 10:**
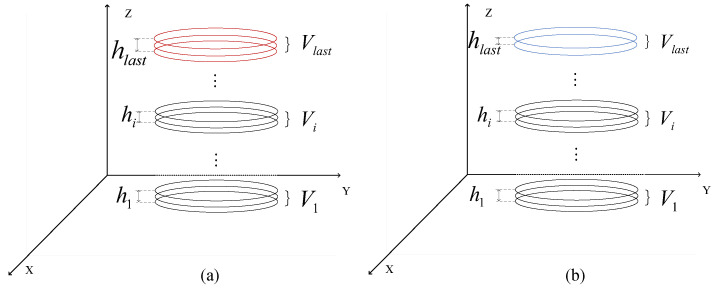
Schematic representation of volume accumulation strategies for sliced point clouds with different layer counts. (**a**) Schematic diagram of volume accumulation when the total number of layers is odd. (**b**) Schematic diagram of volume accumulation when the total number of layers is even. The different colors in the figure are primarily used to distinguish between even/odd indexed slices and to highlight the final cumulative volume for better visual clarity.

When the total number of layers is odd, all layers can be divided into several complete three-layer infinitesimal groups, and Simpson’s rule is directly applied for global accumulation as shown in Figure 10a. When the total number of layers is even, the first n−2 layers still use Simpson’s rule to calculate the volume infinitesimals as illustrated in Figure 10b, while the last two layers use the frustum volume formula for supplementary calculation. The frustum volume formula originates from the differential derivation of pyramid volume and is suitable for calculating the solid volume enclosed by two parallel sections, as expressed in (26).

(26) $$V_{last}=\frac{h_{last}}{3}\left(S_{n-1}+S_{n}+\sqrt{S_{n-1}S_{n}}\right)$$

where $h_{last}=z_{n}-z_{z-1}$ is the height difference of the last two layers. By introducing the geometric mean of the cross-sectional areas, this formula compensates for the poor adaptability of Simpson’s rule to the terminal of even-numbered layers.

Synthesizing the above logic, the final calculation operator for the total volume $V_{total}$ of the part is defined as in (27).

(27) $$V_{\mathrm{total}}=\left\{\begin{array}{cc} \sum_{i=1,3,5,\ldots }^{n-2}\frac{z_{i+2}-z_{i}}{6}\left(S_{i}+4S_{i+1}+S_{i+2}\right), & n\;\mathrm{is}\;\mathrm{odd}\\ \begin{array}{cc} \; & \sum_{i=1,3,5,\ldots }^{n-3}\frac{z_{i+2}-z_{i}}{6}\left(S_{i}+4S_{i+1}+S_{i+2}\right)\\ \; & +\frac{z_{n}-z_{n-1}}{3}\left(S_{n-1}+S_{n}+\sqrt{S_{n-1}S_{n}}\right) \end{array} & ,n\;\mathrm{is}\;\mathrm{even} \end{array}\right.$$

Through the coupling of Simpson’s rule and the frustum volume formula, this paper not only leverages the high-precision calculation advantages of numerical integration for continuously varying sections but also accounts for the discrete physical characteristics of actual scanned layered data. This method effectively solves the engineering challenge of accurately quantifying the volume of irregular pits, and combined with strict data verification steps, ensures the stability of the calculation process and the reliability of the results.

## 5. Experiments and Results Analysis

### 5.1. Experimental Design

#### 5.1.1. Experimental Scenarios

To verify the reconstruction accuracy of the algorithm for complex pit features, this study designed and fabricated a set of simulated pit devices with typical topological defects. The main body of the device is a cylindrical-like structure with a diameter of approximately 25 cm and a depth of approximately 29 cm. Its inner wall is manually attached with irregular protrusion blocks ranging from 1–3 cm or a 5 mm thick sponge layer cut with transverse stripes. These are used to simulate common macro-working conditions in engineering, such as borehole necking and local over-excavation, as well as microscopic tool marks left by mechanical excavation.

**Figure 11:**
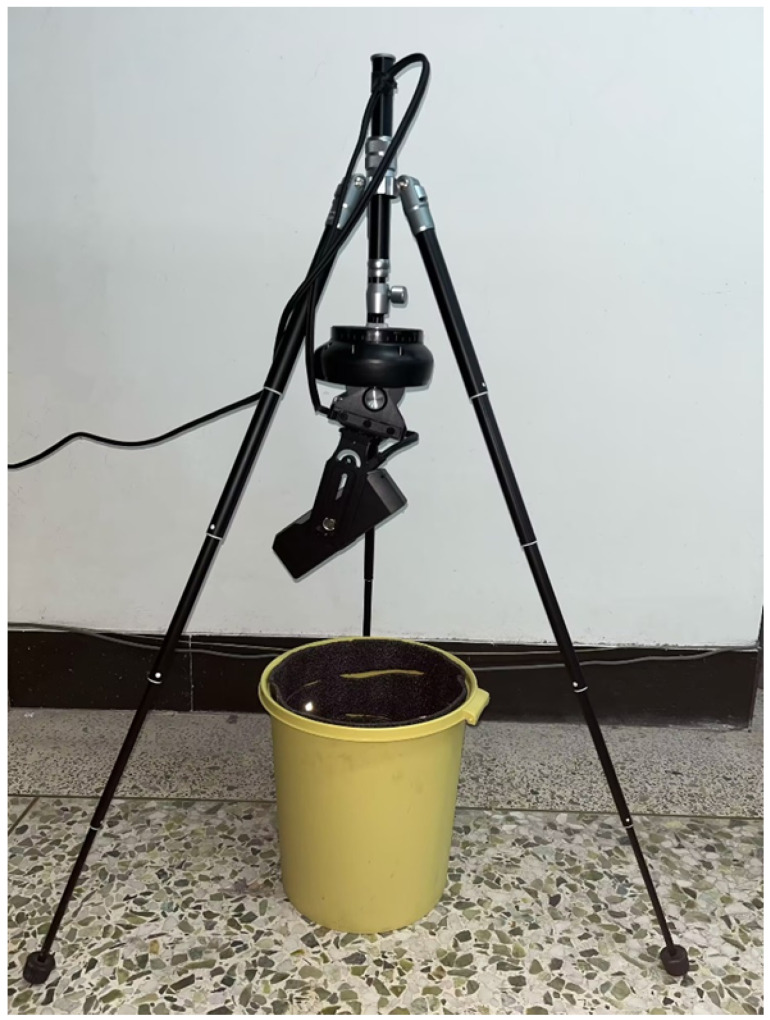
Experimental setup for pit data acquisition.

**Figure 12:**
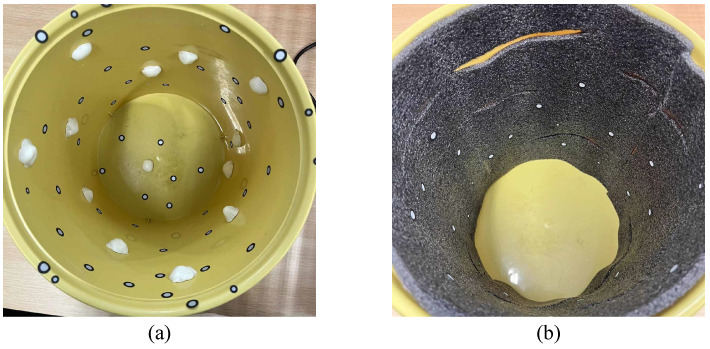
Detailed view of simulated pit defect groups. (**a**) Local Protrusion Group: simulating structural defects, (**b**) Detailed Texture Group: simulating mechanical tool marks.

During the experimental process, a 3D laser scanner was used to perform multi-view circular scanning of the interior of the simulated device to obtain original point clouds containing complex occlusion relationships. The actual model setup scene is shown in Figure 11. Figure 12 shows the specific model types.

#### 5.1.2. Data Acquisition Procedure

The experimental scheme designed in this paper aims to comprehensively evaluate the performance of the volume detection algorithm through a progressive logic of standard model calibration, artificial defect verification, and actual working condition simulation.

To ensure the precision and consistency of data acquisition, this study adopts a high-precision handheld laser scanning system as the core sensing unit. The scanner captures the 3D coordinate information of the pit inner wall in real time by emitting laser lines and receiving their reflected signals.

**Figure 13:**
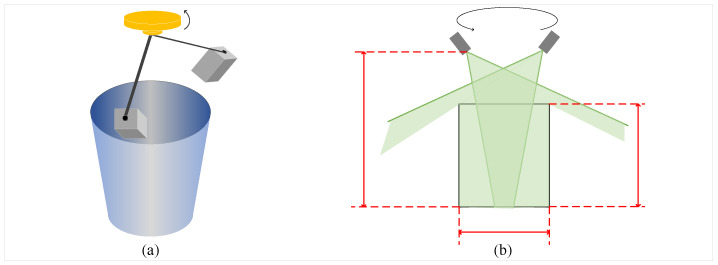
Schematic diagram of experimental setup for pit data acquisition. (**a**) Stereoscopic diagram of the setup. (**b**) Scanning schematic diagram of the setup.

The specific instrument operating principle and scanning path are shown in Figure 13. Figure 13a illustrates the relative spatial position between the scanner and the inner wall of the pit. Through the circumferential rotation and pose adjustment of the sensor above the pit, the perception of the internal geometric morphology of the deep hole is achieved. Figure 13b further describes the coverage range of the scanning system. Through the cross-coverage of multi-path laser projection, the occlusion blind zones caused by complex protrusions on the inner wall can be effectively reduced, ensuring the completeness of 3D coordinate acquisition.

The key instrument performance indicators and parameter settings in the experiment are shown in Table 1.

In the specific data acquisition process, to fully obtain the information of the pit’s inner wall, a circumferential multi-path scanning strategy was adopted. Aiming at the detailed features manually attached to the inner wall of the simulated device, supplementary scanning of occluded areas was prioritized during the scanning process.

All experiments use the physical volume obtained by the water injection calibration method as the ground truth for comparative analysis of the accuracy deviation of the algorithm in this paper.

### 5.2. Experimental Datasets and Test Conditions

A total of three typical simulated working condition data were collected and processed in this experiment, with 5 sets for each data type. Based on the variation in complexity, these data are categorized into the following three groups.

- Smooth Benchmark Group: The original inner wall without added protrusions, used to test the background noise processing capability of the algorithm.
- Local Protrusion Group: Simulates asymmetric and irregular hole wall morphology caused by uneven geological hardness or local collapse. Through irregular protrusion blocks attached to the inner wall, the geometric robustness of the piecewise fitting integration algorithm under extreme asymmetry and high-curvature contours is tested.
- Detailed Texture Group: Simulates periodic tool marks left during the mechanical excavation process. Geometric undulations are generated through transverse incisions on the sponge layer to verify whether the algorithm can accurately extract the core cross-sectional contour through adaptive thinning and smooth fitting in the face of high-frequency detail interference, evaluating the anti-noise capability of area quantization.

**Figure 14:**
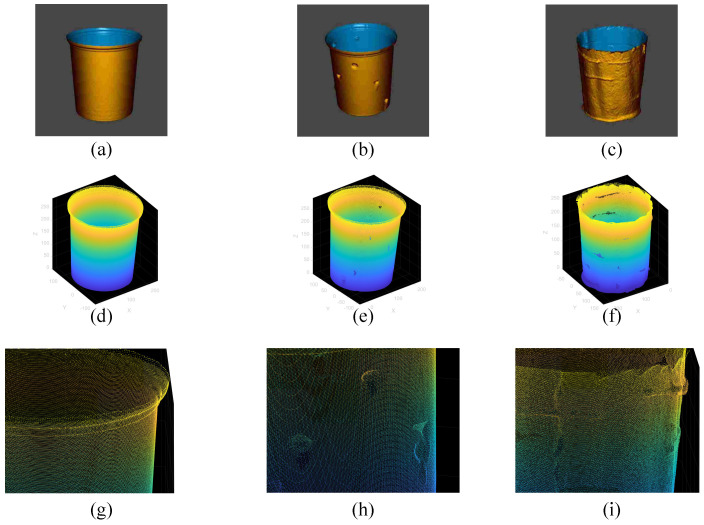
Visualization of the three simulated experimental conditions and their corresponding point cloud data. (**a**) Scanning model of Smooth Benchmark Group. (**b**) Scanning model of Local Protrusion Group. (**c**) Scanning model of Detailed Texture Group. (**d**) Point cloud of Smooth Benchmark Group. (**e**) Point cloud of Local Protrusion Group. (**f**) Point cloud of Detailed Texture Group. (**g**) Partial view of point cloud for Smooth Benchmark Group. (**h**) Partial view of point cloud for Local Protrusion Group. (**i**) Partial view of point cloud for Detail Texture Group. In subfigures (**a**–**c**), the different colors distinguish the inner surface from the outer surface of the model. In subfigures (**d**–**i**), the color gradient represents the height (Z-axis) of the point cloud.

The visualization effects of the above three groups of simulated working conditions are shown in Figure 14. Figure 14a–c display the reconstructed models generated after scanning the physical objects, intuitively presenting the differences between the Smooth Benchmark Group, Local Protrusion Group, and Detailed Texture Group; Figure 14d–f show the corresponding original point cloud models; Figure 14g–i provide local enlarged views of the corresponding point clouds, further demonstrating the subtle differences in each data set, especially the simulated tool marks in the Detailed Texture Group.

The original point cloud scale of each group ranges from 200,000 to 400,000 points, saved in a standard point cloud format with normal information.

### 5.3. Measurement Accuracy and Error Distribution Analysis

#### 5.3.1. Methodology

To verify the impact of point cloud simplification on calculation accuracy, this study compares the volume error rates of the original point clouds and the simplified sparse point clouds.

As illustrated in Table 2, the error rates for both methods remain consistently low, with the maximum absolute difference between them being only 0.09%. This result demonstrates that the simplification process effectively preserves the geometric features of the pits without causing a substantial loss in volume calculation precision. Furthermore significant reduction in data volume leads to a marked improvement in computational efficiency, proving that this preprocessing step ensures both high accuracy and superior time performance.

Following the validation of the reliability of the point cloud preprocessing step, multiple sets of repeated experiments were conducted under various working conditions of differing complexity to further evaluate the comprehensive performance of the proposed piecewise-cubic polynomial fitting and adaptive volume calculation model. For each typical point cloud, multiple tests were performed, and the average values were calculated to eliminate the randomness of individual measurements.

The accuracy of the volume detection algorithm is primarily quantitatively evaluated through the relative volume error δ, which is defined as the percentage of the absolute value of the deviation between the calculated volume $V_{calc}$ output by the algorithm and the physical ground truth $V_{true}$ relative to the ground truth. The specific calculation formula is as in (28).

(28) $$\delta =\frac{\left|V_{calc}-V_{true}\right|}{V_{true}}\times 100$$

According to the experimental data in Table 3, the volume calculation error rates for the three typical working conditions all exhibit extremely high consistency, with the overall error controlled within 0.8%. Specifically, the Smooth Benchmark Group have the lowest average error, maintained between 0.03% and 0.40%; the Detailed Texture Group is disturbed by microscopic geometric undulations, with the error fluctuating slightly between 0.16% and 0.66%; meanwhile, the Local Protrusion Group exhibits significant robustness when facing extremely asymmetric contours, with a maximum error rate of only 0.76%, proving the effectiveness of the differentiated aggregation strategy under extreme working conditions.

Furthermore, statistical analysis indicates that the standard deviations of all groups remain at a low level, and the 95% confidence intervals are narrow. In particular, the Smooth Benchmark Group shows no significant deviation, while the Local Protrusion Group and Detailed Texture Group demonstrate statistically significant errors, confirming both the stability and reliability of the proposed method under varying geometric complexities.

To further evaluate the effectiveness of the proposed method, comparative experiments were conducted using several representative volume calculation approaches, including Delaunay triangulation, Convex Hull, and Alpha Shape. All methods were tested on the same local protrusion datasets under identical preprocessing conditions.Three of the methods for setting parameters are shown in Table 4.

According to the experimental data in Table 5, The convex hull method shows the largest deviation due to its inability to represent concave structures, while the triangulation method suffers from mesh approximation errors. The alpha shape method performs relatively better but still exhibits higher error than the proposed approach. By contrast, the proposed method achieves lower error and improved robustness due to adaptive simplification and piecewise contour fitting.

#### 5.3.2. Error Distribution Analysis

By comparing different working conditions, layer thicknesses, and sliding window parameters, this paper analyzes the algorithm’s applicability and stability. In the sensitivity evaluation of cross-sectional layer thickness, when the layer thickness increases to 2 mm or more, the error increases significantly because the excessive spacing fails to finely characterize the high-frequency geometric changes of the inner wall; although the 0.1 mm layering precision is extremely high, the computational time consumption surges due to the excessive point cloud scale. Therefore, this paper selects 0.5 mm as the optimal layer spacing to balance reconstruction accuracy and processing efficiency. The impact analysis of sliding window parameters indicates that the piecewise adjustment mechanism based on Algorithm 2 is the core of ensuring data quality. As shown in Figure 9, the adoption of a wide window in the early stable segment effectively enhances the suppression of random noise, while switching to a narrow window in the later gradual segment significantly improves the response sensitivity to the intense evolutionary trends of geometric morphology. This dynamic parameter setting ensures that the area sequence can still accurately restore true physical features at complex abrupt change positions, providing a reliable data guarantee for the final high-precision volume quantification.

## 6. Conclusions

This study proposed a 3D perception-based volume measurement framework for irregular soil compaction pits using adaptive point cloud simplification and layered reconstruction. The method integrates pose normalization, density-adaptive point cloud thinning, morphology-aware contour fitting, and a hybrid Simpson–frustum integration strategy to improve robustness under non-uniform sampling and complex boundary conditions. Experimental results on multiple simulated pit scenarios demonstrated that the proposed approach maintained high geometric fidelity after simplification while achieving reliable volume estimation, with an average relative error below 0.8%. These results indicate that the framework provides an effective and accurate solution for automated pit volume quantification in subgrade compaction quality assessment. Beyond compaction pit assessment, the hierarchical projection–integration architecture also demonstrates strong extensibility for other close-range 3D reconstruction scenarios, including heritage documentation, tunnel erosion monitoring, and high-precision industrial inspection.

## 7. Future Work

Although our algorithm has achieved ideal volume calculation results in artificial defect scenarios, there is still room for improvement in other contexts. For instance, in bottom areas of holes with extremely high depth-to-diameter ratios, challenges in volume compensation arising from low point cloud integrity may be encountered. Therefore, future research will focus on improving engineering applicability by geometric-topological completion of incomplete point cloud regions in complex hole bottoms and curvature-aware adaptive volume compensation algorithms.

Although the current study validates the proposed framework using simulated pit devices with controlled geometric defects, the algorithm is designed to address irregular compaction pits encountered in engineering practice. The simulated datasets were intentionally constructed to reproduce typical geometric conditions observed in roadbed compaction inspection, including local protrusions, asymmetric wall deformation, and fine-scale surface texture variations. However, real engineering environments may introduce additional uncertainties, such as uneven illumination, loose soil interference, partial occlusion caused by surrounding structures, and variable scanning accessibility. These factors may affect both point cloud completeness and registration stability.

Future work will focus on validating the proposed method using field-acquired roadbed compaction pits and construction-site datasets. In particular, comparisons between simulated and real-world measurements will be conducted to further evaluate robustness and operational applicability.

In addition, future research will investigate the integration of image-assisted reconstruction strategies to further reduce measurement uncertainty caused by scanning-device limitations, illumination variation, and local surface reflectance differences. By incorporating complementary image information, the framework is expected to improve feature continuity and enhance geometric recovery in regions where point cloud acquisition is incomplete or unstable.

Future comparative studies will also consider a broader range of evaluation factors, including acquisition efficiency, registration robustness, computational complexity, and multi-environment adaptability. These expanded comparisons will provide a more comprehensive assessment of the proposed framework relative to existing volumetric measurement approaches.

## Figures and Tables

**Table 1:** Scanning equipment specifications.

Parameter	Specification
Light Source	NIR Structured Light
Accuracy	Up to 0.02 mm
3D Resolution	0.05 mm–2.0 mm
Scanning Speed	Up to 20 frames per second (fps)
Object Size Range	10 mm × 10 mm × 10 mm to 2000 mm × 2000 mm × 2000 mm
Alignment Modes	Geometry/Marker/Texture
Rotation Speed	2°–6° per second

**Table 2:** Performance evaluation of point cloud simplification.

Dataset	Raw Point	Raw Error	Simplified	Simplified	Absolute
	Cloud Count	Rate	Point Count	Error Rate	Difference
Dataset 1	259,723	0.104%	82,734	0.122%	0.018%
Dataset 2	287,578	0.192%	93,353	0.235%	0.043%
Dataset 3	210,121	0.048%	81,396	0.079%	0.031%
Dataset 4	339,817	0.345%	79,802	0.400%	0.055%
Dataset 5	355,908	0.110%	80,275	0.069%	0.041%
Dataset 6	293,757	0.053%	79,757	0.026%	0.027%
Dataset 7	319,704	0.223%	83,651	0.311%	0.088%
Dataset 8	278,390	0.036%	80,628	0.063%	0.027%
Dataset 9	226,391	0.213%	86,106	0.285%	0.072%
Dataset 10	308,259	0.149%	78,362	0.125%	0.024%

**Table 3:** Volume calculation and error analysis of different point cloud types with statistical metrics.

Point Cloud Type	Calculated Volume (cm^3^)	Actual Volume (cm^3^)	Error Rate	Average Error	Std Dev	95% CI	*p*-Value
Smooth Benchmark 1	10,740.502	10,783.6	0.40%				
Smooth Benchmark 2	10,791.041	10,783.6	0.07%				
Smooth Benchmark 3	10,786.376	10,783.6	0.03%	0.165%	0.145%	[−0.014%, 0.346%]	0.063
Smooth Benchmark 4	10,804.524	10,783.6	0.19%				
Smooth Benchmark 5	10,798.536	10,783.6	0.14%				
Local Protrusion 1	10,774.536	10,758.5	0.15%				
Local Protrusion 2	10,840.075	10,758.5	0.76%				
Local Protrusion 3	10,711.958	10,758.5	0.43%	0.433%	0.229%	[0.149%, 0.719%]	0.013
Local Protrusion 4	10,702.845	10,758.5	0.52%				
Local Protrusion 5	10,791.543	10,758.5	0.31%				
Detailed Texture 1	9097.204	9148.3	0.56%				
Detailed Texture 2	9194.671	9148.3	0.51%				
Detailed Texture 3	9087.597	9148.3	0.66%	0.469%	0.191%	[0.261%, 0.734%]	0.004
Detailed Texture 4	9106.358	9148.3	0.46%				
Detailed Texture 5	9162.808	9148.3	0.16%				

**Table 4:** Parameter settings for the comparative reconstruction methods.

Delaunay Triangulation		Convex Hull		Alpha Shape	
Parameter	Value	Parameter	Value	Parameter	Value
Maximum edge length	6 mm	Hull mode	Global	Alpha radius	6 mm
Triangle area threshold	30 mm^2^	Voxel downsample	2 mm	Neighbor count	12
Neighbor search radius	5 mm	Statistical neighbor	15	Minimum cluster size	100 pts
Hole filling radius	5 mm	Std ratio	1.2	Hole filling	Enabled
Surface smoothing	2 iterations	Boundary closure	Enabled	Boundary smoothing	2 iterations

**Table 5:** Comparison of different volume calculation methods on local protrusion point clouds.

Method	Sample	Calculated Volume (cm^3^)	Actual Volume (cm^3^)	Error Rate	Average Error
	Local 1	10,801.6	10,758.5	0.40%	
	Local 2	10,861.2	10,758.5	0.96%	
Delaunay Triangulation	Local 3	10,705.3	10,758.5	0.49%	0.599%
	Local 4	10,689.4	10,758.5	0.64%	
	Local 5	10,812.7	10,758.5	0.50%	
	Local 1	10,832.9	10,758.5	0.69%	
	Local 2	10,902.4	10,758.5	1.34%	
Convex Hull	Local 3	10,801.5	10,758.5	0.40%	0.791%
	Local 4	10,846.3	10,758.5	0.82%	
	Local 5	10,835.1	10,758.5	0.71%	
	Local 1	10,792.4	10,758.5	0.32%	
	Local 2	10,852.6	10,758.5	0.87%	
Alpha Shape	Local 3	10,718.9	10,758.5	0.37%	0.507%
	Local 4	10,698.6	10,758.5	0.56%	
	Local 5	10,803.5	10,758.5	0.42%	

## Data Availability

The authors declare that all data supporting the findings of this study are available within the article.
